# Clinical characteristics and predictors of prolonged hospitalization in patients with cosmetic botulinum toxin poisoning: a retrospective cohort study

**DOI:** 10.3389/fmed.2026.1836545

**Published:** 2026-06-22

**Authors:** Yu-quan Chen, Yi-fan Ye, Mei-wen Xie, Yu-qiang Lin, Zhi-qian Yang, Zhi Wang

**Affiliations:** Department of Occupational Diseases and Poisoning, Guangzhou Occupational Disease Prevention and Treatment Hospital (Guangzhou Twelfth People’s Hospital), Guangzhou, China

**Keywords:** antitoxin therapy, cosmetic botulinum toxin poisoning, iatrogenic botulism, nomogram, prolonged hospitalization

## Abstract

**Background:**

The increasing cosmetic use of botulinum toxin has been accompanied by poisoning events related to inappropriate administration, including excessive dosing, unapproved or unverifiable products, self-injection, and procedures performed outside regulated medical settings. However, the inpatient clinical profile and predictors of prolonged hospitalization remain insufficiently defined. This study aimed to describe the clinical characteristics of cosmetic botulinum toxin poisoning and identify factors associated with longer hospital stay.

**Methods:**

We conducted a retrospective cohort study of patients hospitalized for cosmetic botulinum toxin poisoning at Guangzhou Twelfth People’s Hospital between August 2024 and January 2025. Demographic data, injection-related characteristics, clinical manifestations, antitoxin treatment, hospitalization duration, and available clinical outcomes were extracted from medical records. Patients were divided into a short-stay group (<7 days) and a prolonged-stay group (≥7 days). Univariate analyses and multivariable Cox regression were performed to identify factors associated with hospitalization duration. A nomogram was developed to estimate length of stay.

**Results:**

A total of 145 patients were included, with a median age of 37 years; 97.93% were female. The most common injection sites were the forehead (26.35%), periocular region (16.77%), and jaw (11.98%). The most frequent manifestations were dizziness (89.66%), dysphagia (85.52%), blurred vision (75.17%), ptosis (68.28%), and slurred speech (24.83%). In multivariable Cox regression, duration of antitoxin therapy, dysphagia, and slurred speech were independently associated with prolonged hospitalization. A nomogram based on these variables showed acceptable calibration performance (MSE = 1.302; MAE = 1.029). No in-hospital deaths occurred.

**Conclusion:**

Cosmetic botulinum toxin poisoning is characterized mainly by cranial nerve-related and bulbar manifestations. Dysphagia, slurred speech, and antitoxin treatment duration were independently associated with prolonged hospitalization. The nomogram may support early risk stratification and inpatient management, although external validation is needed.

## 1 Introduction

Botulinum toxin is one of the most potent known biological toxins produced by *Clostridium botulinum* under anaerobic conditions (1). By cleaving soluble N-ethylmaleimide-sensitive factor attachment protein receptor (SNARE) proteins and inhibiting acetylcholine release at the neuromuscular junction, botulinum toxin causes cranial nerve palsies, bulbar dysfunction, and progressive descending flaccid paralysis, which may lead to respiratory compromise in severe cases (1, 2). Although botulinum toxin has established therapeutic and cosmetic applications (17), systemic poisoning may occur after inappropriate administration, including excessive dosing, use of unapproved or unverifiable products, self-injection, or procedures performed outside regulated medical settings (3–10).

In recent years, cosmetic botulinum toxin injection has become increasingly common, particularly among women seeking facial esthetic procedures. Alongside this trend, cosmetic botulinum toxin poisoning has emerged as a clinically important problem (3, 4, 7, 8, 11). Unlike classic foodborne botulism, cosmetic botulinum toxin poisoning usually occurs after an elective cosmetic procedure, and patients often seek care for cranial nerve-related and bulbar symptoms such as dysphagia, blurred vision, ptosis, and dysarthria (3, 6, 8, 11–13). Because these patients are usually managed in hospital settings, factors associated with prolonged hospitalization are of direct clinical relevance.

Previous reports have mainly focused on case descriptions, outbreaks, emergency treatment, or public health responses rather than inpatient risk stratification (3, 4, 6–8, 11, 13–16). Data on the inpatient clinical profile of cosmetic botulinum toxin poisoning and the determinants of hospital stay remain limited. Therefore, in this retrospective cohort study, we analyzed 145 hospitalized patients with cosmetic botulinum toxin poisoning to characterize their clinical presentations, identify predictors of prolonged hospitalization, and develop a practical model for estimating length of stay. These findings may support early risk stratification and improve inpatient management in this increasingly encountered toxic exposure.

## 2 Methods

### 2.1 Study design and setting

This retrospective cohort study included consecutive patients admitted to Guangzhou Twelfth People’s Hospital for cosmetic botulinum toxin poisoning between August 2024 and January 2025. Hospitalized cases were identified by searching the electronic medical record system and the departmental poisoning registry for patients admitted with suspected or confirmed botulinum toxin poisoning during the study period. Search terms and diagnostic labels included “botulinum toxin poisoning,” “botulism,” “cosmetic botulinum toxin injection,” and related discharge diagnoses. Medical records were then reviewed manually to confirm eligibility. A total of 149 cases were initially screened, and 145 patients met the final eligibility criteria and were included in the analysis.

### 2.2 Ethical approval

This study was approved by the Medical Ethics Committee of Guangzhou Occupational Disease Prevention and Treatment Hospital (Guangzhou Twelfth People’s Hospital). Patient records were reviewed retrospectively, all personal identifiers were removed, and data confidentiality was strictly maintained. Because this study used anonymized retrospective clinical data, the requirement for written informed consent was waived by the ethics committee. Ethical procedures were conducted in accordance with institutional requirements and the Declaration of Helsinki.

### 2.3 Case definition and eligibility criteria

For this study, a case of cosmetic botulinum toxin poisoning was defined as a hospitalized patient with: (1) a documented history of botulinum toxin injection for cosmetic purposes within 30 days before symptom onset; (2) compatible clinical manifestations after injection, particularly cranial nerve-related or bulbar symptoms such as dizziness, blurred vision, ptosis, dysphagia, dysarthria/slurred speech, hoarseness, neck weakness, dyspnea, or shortness of breath; (3) a clinical diagnosis of botulinum toxin poisoning made by the treating physicians; and (4) no alternative diagnosis that better explained the clinical presentation. Because laboratory confirmation of botulinum toxin was not routinely available in this retrospective cohort, case identification was based on exposure history, compatible clinical manifestations, clinical diagnosis, and exclusion of other forms of botulism or alternative causes.

A 30-day exposure window was selected to capture clinically plausible delayed systemic manifestations after cosmetic botulinum toxin injection while reducing the likelihood of including unrelated neurological or respiratory conditions. This time frame also provided a practical and reproducible criterion for retrospective case identification.

Patients were eligible if they met all of the following criteria: (1) met the above case definition; (2) were hospitalized for evaluation and treatment of cosmetic botulinum toxin poisoning; and (3) had sufficient clinical data for analysis. Patients were excluded if they received botulinum toxin for clearly therapeutic or non-cosmetic indications, had suspected or confirmed foodborne botulism, wound botulism, or other non-cosmetic forms of botulism, did not meet the case definition, or had incomplete clinical records preventing analysis.

The purpose and setting of injection were determined from medical records and patient self-report. Injections were classified as cosmetic when the documented or reported purpose was facial esthetic improvement, wrinkle reduction, jawline slimming, body contouring, or other esthetic purposes. Cases with clearly therapeutic indications, such as dystonia, spasticity, strabismus, or hyperhidrosis, were excluded. For injection sites that could be used for either cosmetic or therapeutic purposes, such as axilla, lumbar region, or limbs, cases were retained only when the available record indicated a cosmetic purpose; otherwise, they were excluded.

### 2.4 Data collection

Clinical data were extracted from the electronic medical records of eligible patients. The following variables were collected: age, sex, injection dose, number of injections, number of injection sites, latency from injection to symptom onset, timing of antitoxin initiation, duration of antitoxin therapy, hospitalization duration, discharge status, and major clinical manifestations. Clinical symptoms analyzed in this study included dizziness, unconsciousness, blurred vision, slurred speech, hoarseness, dysphagia, nausea and vomiting, cough with sputum, chest tightness, dyspnea, shortness of breath, neck weakness, and ptosis.

Information on toxin preparation, product source, brand, licensing status, and reported toxin type was extracted when documented. However, because many patients could not provide complete product packaging, purchase channels, or verifiable documentation, product formulation and licensing status could not be reliably confirmed for all cases. Therefore, these variables were summarized descriptively when available but were not included in the multivariable model.

Although nausea and vomiting are not typical hallmark manifestations of iatrogenic botulism, they were recorded as presenting symptoms to provide a comprehensive description of the initial clinical spectrum and to account for alternative or overlapping clinical presentations at admission. The purpose and setting of injection were determined from medical records and patient self-report. Injections were classified as cosmetic when the documented or reported purpose was facial esthetic improvement, wrinkle reduction, jawline slimming, body contouring, or other esthetic purposes. Cases with clearly therapeutic indications, such as dystonia, spasticity, strabismus, or hyperhidrosis, were excluded. For injection sites that could be used for either cosmetic or therapeutic purposes, such as axilla, lumbar region, or limbs, cases were retained only when the available record indicated a cosmetic purpose; otherwise, they were excluded.

### 2.5 Antitoxin treatment

Antitoxin therapy was administered according to the institutional treatment protocol, the package insert, and the treating physicians’ clinical judgment. The antitoxin used in this cohort was botulinum antitoxin type A injection, a licensed therapeutic product approved in China (National Medical Products Administration approval number: S10820152), rather than an investigational product. According to the package insert available at our institution, this preparation is an equine-derived monovalent botulinum antitoxin produced from plasma obtained after immunization of horses with botulinum toxin type A or toxoid, followed by pepsin digestion and purification. The product was supplied as 10,000 IU per vial, with each vial containing 4 mL of botulinum antitoxin type A.

The approved indication is prevention and treatment of type A botulism. According to the package insert, the recommended therapeutic dose is 10,000–20,000 IU per administration by intramuscular injection or intravenous infusion, with repeated administration approximately every 12 h when clinically indicated according to disease severity and symptom progression. In this study, “duration of antitoxin therapy” was defined as the number of calendar days on which botulinum antitoxin type A injection was administered, rather than the total number of doses. Information on daily dose, number of administrations, and cumulative dose was extracted when available. However, cumulative antitoxin dose and product-specific serum half-life were not consistently documented in the retrospective medical records. In addition, serum half-life was not specified in the package insert available at our institution. Therefore, these variables could not be reliably incorporated into the multivariable model.

### 2.6 Outcome and statistical analysis

The primary outcome was hospitalization duration. Because the median hospital stay in the cohort was 7 days, patients were categorized into a short-stay group (<7 days) and a prolonged-stay group (≥7 days) for univariate comparison. Clinical outcomes during hospitalization and available follow-up information after discharge were summarized descriptively.

Statistical analyses were performed using SPSS version 25.0, and figures were generated in R. Categorical variables are presented as counts and percentages. Continuous variables were summarized as mean ± standard deviation when normally distributed or as median and interquartile range when non-normally distributed. Group comparisons were conducted using the independent-samples *t* test, Mann–Whitney U test, chi-square test, or Fisher’s exact test, as appropriate. Variables with clinical relevance or a univariate *p* value <0.10 were entered into a multivariable Cox regression model to identify independent factors associated with hospitalization duration. Hazard ratios (HRs) and 95% confidence intervals (CIs) were calculated. In the Cox regression model, discharge was treated as the event; therefore, an HR < 1 indicated a lower discharge rate over time and a tendency toward longer hospitalization. A two-sided *p* value <0.05 was considered statistically significant. Based on the final multivariable model, a nomogram was constructed to estimate length of stay, and calibration was summarized using mean square error (MSE) and mean absolute error (MAE).

## 3 Results

### 3.1 Baseline characteristics

**Figure 1:**
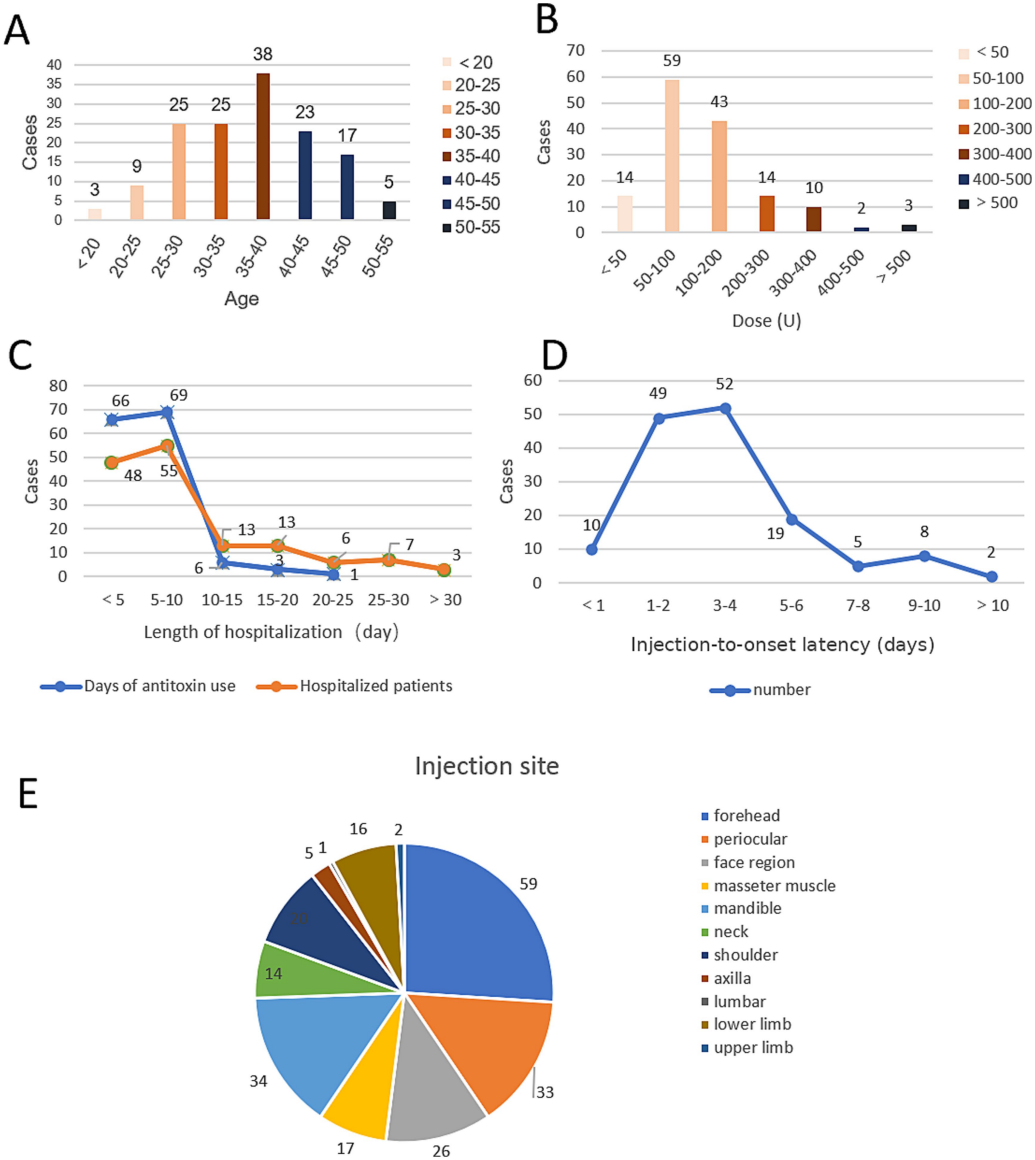
Distribution of clinical characteristics among 145 patients with cosmetic botulinum toxin poisoning. **(A)** Age distribution. **(B)** Injection dose distribution. **(C)** Distribution of hospitalization duration and duration of antitoxin therapy. **(D)** Injection-to-onset latency. **(E)** Injection site distribution.

A total of 145 patients were included in the final analysis. Patients ranged in age from 12 to 55 years, with a median age of 37 years, and females accounted for 97.93% of the cohort. One patient was 12 years old. This case was retained because the patient had a documented cosmetic botulinum toxin exposure and compatible clinical manifestations requiring hospitalization. However, cosmetic botulinum toxin use in children or adolescents is outside standard adult cosmetic indications and is not recommended. The most frequent injection sites were the forehead (26.35%), periocular region (16.77%), and jaw (11.98%). Most patients received a single injection session (93.1%), whereas 6.9% received two injections. Regarding injection dose, 100 U was the most common dose (35.2%), followed by 200 U (22.1%). The median hospitalization duration was 7 days (IQR, 5–12). The median latency from injection to symptom onset was 3 days (IQR, 2–4). The median time from symptom onset to antitoxin initiation was 7 days (IQR, 5–11). The distributions of age, injection dose, hospitalization duration, latency from injection to symptom onset, and injection site are shown in Figure 1. Many patients reported receiving injections under non-standard circumstances, including suspected counterfeit, unapproved, or unverifiable botulinum toxin products; injections performed in settings without verifiable medical qualification; self-injection; friend-assisted injection; excessive dosing; or injections at multiple sites. Because product brand, package verification, formal labeling information, and provider qualification were not consistently available in the retrospective records, cases could not be reliably stratified into labeling-indicated versus off-label cosmetic use for all patients. Patients receiving botulinum toxin for clearly therapeutic or non-cosmetic indications were excluded from the final cohort. For injection sites that may overlap with therapeutic indications, such as axilla, lumbar region, or limbs, available records indicated that the reported purpose was cosmetic.

### 3.2 Clinical manifestations

**Figure 2:**
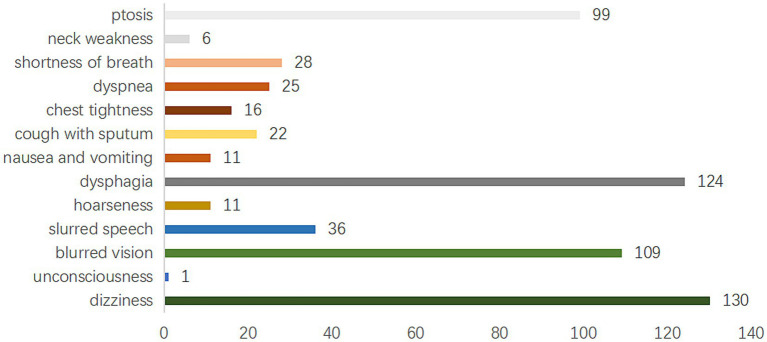
Clinical manifestations in 145 patients with cosmetic botulinum toxin poisoning.

Thirteen clinical features were analyzed. The most common symptoms were dizziness in 130 patients (89.66%), dysphagia in 124 (85.52%), blurred vision in 109 (75.17%), ptosis in 99 (68.28%), and slurred speech in 36 (24.83%). Additional manifestations included shortness of breath in 28 patients (19.31%), dyspnea in 25 (17.24%), cough with sputum in 22 (15.17%), chest tightness in 16 (11.03%), hoarseness in 11 (7.59%), nausea and vomiting in 11 (7.59%), neck weakness in 6 (4.14%), and unconsciousness in 1 patient (0.69%). These findings indicate that cranial nerve-related and bulbar symptoms predominated in this cohort. The distribution of clinical manifestations is shown in Figure 2.

### 3.3 Univariate analysis according to hospitalization duration

To explore factors associated with prolonged hospitalization, patients were divided into a short-stay group (<7 days, *n* = 66) and a prolonged-stay group (≥7 days, *n* = 79). Univariate analysis showed that injection dose (*p* = 0.049), duration of antitoxin therapy (*p* < 0.001), latency from injection to symptom onset (*p* = 0.020), and dysphagia (*p* = 0.002) differed significantly between groups. Slurred speech showed a borderline association (*p* = 0.090) and was retained for multivariable analysis because of its clinical relevance. No significant between-group differences were observed for age, number of injection sites, number of injections, blurred vision, ptosis, or most other symptoms. The univariate comparisons according to hospitalization duration are summarized in Table 1.

**Table 1:** Univariate comparisons according to hospitalization duration in 145 patients with cosmetic botulinum toxin poisoning.

Variable	<7 days (*n* = 66)	≥7 days (*n* = 79)	Statistic	*p* value
Age, years	36.47 (29.19, 43.75)	36.87 (28.10, 45.64)	*t* = −0.298	0.766
Number of injection sites	1 (1, 2)	1 (1, 2)	*t* = 0.162	0.872
Number of injections	1 (1, 1)	1 (1, 1)	*t* = 1.615	0.109
Injection dose, U	100 (100, 200)	130 (100, 200)	*t* = −1.984	0.049
Time to antitoxin initiation, days from symptom onset	7 (5, 11)	7 (5, 11)	*t* = 1.655	0.100
Duration of antitoxin therapy, days	4 (4, 8)	8 (4, 8)	*t* = −9.733	<0.001
Latency from injection to symptom onset, days	3 (2, 4)	3 (2, 4)	*t* = 2.364	0.020
Female, *n* (%)	63 (95.5%)	79 (100%)	χ^2^ = 3.667	0.092
Dizziness, *n* (%)	61 (92.4%)	69 (87.3%)	χ^2^ = 1.002	0.317
Unconsciousness, *n* (%)	1 (1.5%)	0 (0%)	χ^2^ = 1.205	0.455
Blurred vision, *n* (%)	50 (75.8%)	59 (74.7%)	χ^2^ = 0.022	0.881
Slurred speech, *n* (%)	12 (18.2%)	24 (30.4%)	χ^2^ = 2.867	0.090
Hoarseness, *n* (%)	5 (7.6%)	6 (7.6%)	χ^2^ = 0.001	0.997
Dysphagia, *n* (%)	50 (75.8%)	74 (93.7%)	χ^2^ = 9.316	0.002
Nausea and vomiting, *n* (%)	5 (7.6%)	6 (7.6%)	χ^2^ = 0.001	0.997
Cough with sputum, *n* (%)	9 (13.6%)	13 (16.5%)	χ^2^ = 0.222	0.637
Chest tightness, *n* (%)	6 (9.1%)	10 (12.7%)	χ^2^ = 0.466	0.495
Dyspnea, *n* (%)	8 (12.1%)	17 (21.5%)	χ^2^ = 2.226	0.136
Shortness of breath, *n* (%)	12 (18.2%)	16 (20.3%)	χ^2^ = 0.099	0.753
Neck weakness, *n* (%)	2 (3.0%)	4 (5.1%)	χ^2^ = 0.375	0.689
Ptosis, *n* (%)	44 (66.7%)	55 (69.6%)	χ^2^ = 0.145	0.704

### 3.4 Multivariable Cox regression

Variables selected from the univariate analysis were entered into a multivariable Cox regression model. Duration of antitoxin therapy (HR 0.787, 95% CI 0.733–0.840, *p* < 0.010), dysphagia (HR 0.558, 95% CI 0.338–0.920, *p* = 0.022), and slurred speech (HR 0.643, 95% CI 0.427–0.967, *p* = 0.034) remained independently associated with hospitalization duration. In the context of discharge as the event, lower HRs indicate a lower discharge rate over time and therefore a tendency toward longer hospitalization. The final multivariable Cox regression model is shown in Table 2.

**Table 2:** Multivariable Cox regression analysis for hospitalization duration in 145 patients with cosmetic botulinum toxin poisoning.

Variable	HR (95%CI)	*p*
Duration of antitoxin therapy, days	0.787 (0.733–0.840)	<0.010
Dysphagia	0.558 (0.338–0.920)	0.022
Slurred speech	0.643 (0.427–0.967)	0.034

### 3.5 Antitoxin treatment, supportive management, and clinical outcomes

All patients who received antitoxin therapy were treated with botulinum antitoxin type A injection. The product used at our institution was a licensed equine-derived monovalent type A antitoxin, supplied as 10,000 IU per vial. No heptavalent botulinum antitoxin or investigational antitoxin product was used in this cohort. The median duration of antitoxin therapy was 7 days (IQR, 4–8). Because cumulative antitoxin dose was not consistently recorded in the retrospective medical records, this variable could not be reliably incorporated into the multivariable model.

Supportive management included close monitoring of respiratory status, aspiration prevention, and nutritional support when clinically indicated. Only a small number of patients required respiratory or nutritional support during hospitalization. Because detailed supportive-care variables were infrequent and not consistently documented in a standardized format for all patients, they were summarized descriptively and were not included in the prediction model.

No deaths were recorded during hospitalization. Based on available discharge records and post-discharge information collected as part of this retrospective study, no deaths were reported. However, systematic long-term follow-up was not available for all patients.

### 3.6 Nomogram for length-of-stay prediction

**Figure 3:**
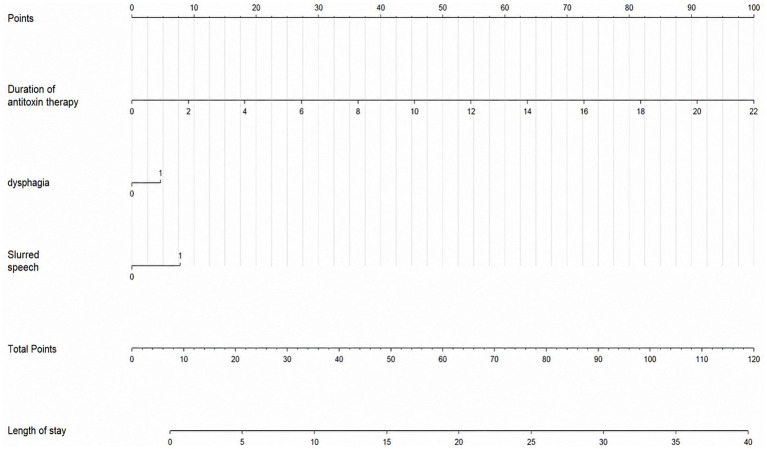
Nomogram for predicting hospitalization duration in patients with cosmetic botulinum toxin poisoning.

**Figure 4:**
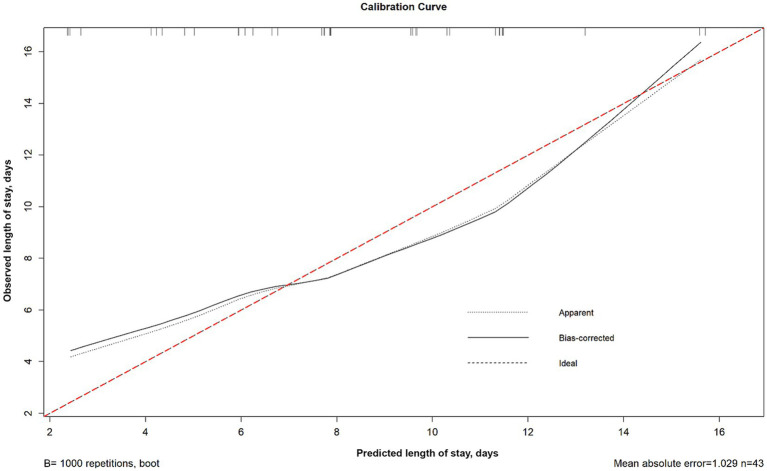
Calibration curve of the nomogram predicting hospitalization duration.

A nomogram incorporating duration of antitoxin therapy, dysphagia, and slurred speech was developed to estimate hospitalization duration. For example, a patient receiving antitoxin for 6 days, with dysphagia but without slurred speech, would have a total score of approximately 32.4, corresponding to an estimated hospitalization duration of about 8 days. Calibration analysis showed acceptable model performance, with MSE = 1.302 and MAE = 1.029. The calibration curve showed reasonable agreement between predicted and observed hospitalization duration (Figures 3, 4).

## 4 Discussion

In this retrospective cohort of 145 hospitalized patients with cosmetic botulinum toxin poisoning, we found that cranial nerve-related and bulbar manifestations were highly prevalent, particularly dizziness, dysphagia, blurred vision, ptosis, and slurred speech. More importantly, duration of antitoxin therapy, dysphagia, and slurred speech were independently associated with hospitalization duration. These findings suggest that bulbar involvement is clinically relevant in identifying patients likely to require longer inpatient care.

The demographic profile of this cohort was notable for a strong female predominance and a median age of 37 years, reflecting the real-world patient population undergoing cosmetic botulinum toxin injection. The most common injection sites were facial regions, including the forehead, periocular area, and jaw, which is consistent with cosmetic practice patterns. Although toxin exposure originated from local injection, the subsequent clinical picture was systemic rather than site-limited, with a high prevalence of dysphagia, blurred vision, ptosis, and slurred speech. This pattern is compatible with the known neurophysiological mechanism of botulinum toxin, which disrupts cholinergic neuromuscular transmission and preferentially manifests through cranial nerve and bulbar dysfunction (1, 2, 15, 16).

Dysphagia emerged as an independent predictor of prolonged hospitalization. This finding is clinically plausible because swallowing dysfunction reflects bulbar muscle involvement and may increase the need for close monitoring, supportive care, aspiration prevention, and nutritional management. In our cohort, dysphagia was present in 93.7% of patients in the prolonged-stay group compared with 75.8% in the short-stay group, supporting its relevance as a marker of more burdensome inpatient management (3, 4, 7, 8, 11).

Slurred speech was also independently associated with longer hospitalization. Similar to dysphagia, dysarthria likely reflects more extensive cranial nerve involvement and more severe neuromuscular impairment. Although its univariate association was borderline, retaining this variable in the multivariable model was justified by both clinical importance and its subsequent independent association in Cox regression. In practical terms, the coexistence of dysphagia and slurred speech may help clinicians identify patients at an early stage who are less likely to recover rapidly and who may require more intensive observation.

Duration of antitoxin therapy was another independent factor associated with hospitalization duration. This variable likely reflects disease severity and ongoing treatment needs rather than a purely causal treatment effect. Patients requiring longer courses of antitoxin therapy may have had more severe systemic involvement, delayed recovery, or greater symptom burden. Because antitoxin neutralizes circulating toxin but does not reverse toxin already bound at the neuromuscular junction, prompt administration remains essential once botulism is clinically suspected (18).

The relatively long interval between symptom onset and antitoxin initiation may be explained by several factors. Early symptoms after cosmetic botulinum toxin exposure may be nonspecific, such as dizziness, blurred vision, mild swallowing discomfort, or fatigue, and patients may initially attribute these symptoms to local injection effects or transient discomfort. In addition, some patients received cosmetic injections outside regulated medical settings or could not provide reliable product information, which may have delayed recognition of systemic botulinum toxin poisoning and referral to a specialized poisoning center. These findings highlight the need for earlier clinical suspicion, prompt neurological and respiratory assessment, and timely antitoxin administration when botulism is clinically suspected.

In this context, standardized management refers to early recognition of suspected botulism, careful assessment of cranial nerve, bulbar, respiratory, and autonomic manifestations, prompt consultation regarding antitoxin therapy, timely antitoxin administration when indicated, aspiration prevention, nutritional support, and close monitoring for respiratory deterioration. Such a structured approach may help reduce delays in treatment and improve inpatient management for patients with cosmetic botulinum toxin poisoning.

The present nomogram was developed as a practical tool for inpatient risk stratification rather than as a highly complex predictive algorithm. Because it is based on readily available clinical variables, it may help physicians anticipate resource use, counsel patients, and identify those requiring closer monitoring during hospitalization. However, the model should be interpreted cautiously because it was derived from a single-center retrospective cohort and has not undergone external validation.

This study has several limitations. First, its retrospective design introduces the possibility of selection bias and residual confounding. Second, this was a single-center study, which may limit generalizability. Third, product formulation, toxin source, brand, licensing status, and exact toxin type were not included in the model because these variables were incompletely and inconsistently documented in the retrospective records. In many cases, exposure information depended on patient self-report, and packaging or purchase channels could not be verified. Including these variables in the model could have introduced substantial misclassification bias. Fourth, cumulative antitoxin dose and detailed treatment intensity were not consistently available in all records. Finally, systematic long-term follow-up was limited, and external validation was not performed. Future multicenter prospective studies with standardized exposure assessment, treatment documentation, and follow-up are needed to confirm these findings and refine prediction tools for cosmetic botulinum toxin poisoning. In addition, labeling indication, provider qualification, product authenticity, and package verification could not be confirmed for all patients because many exposures occurred outside regulated medical settings or involved self-purchased or unverifiable products.

## 5 Conclusion

Cosmetic botulinum toxin poisoning in hospitalized patients is characterized predominantly by bulbar and other cranial nerve-related manifestations. Dysphagia, slurred speech, and duration of antitoxin therapy were independently associated with prolonged hospitalization. These findings may support early clinical risk stratification and more individualized inpatient management. Prompt recognition and timely antitoxin administration remain important in suspected cases. The proposed nomogram provides a preliminary tool for estimating hospital stay, but further external validation and systematic long-term follow-up are required before broader clinical application.

## Data Availability

The anonymized data supporting the conclusions of this article will be made available by the corresponding author upon reasonable request, subject to institutional and ethical requirements.
